# Mucosamin Spray for the Prevention of Oral Mucositis in Cancer Patients Receiving Chemotherapy: A Retrospective Cohort Study

**DOI:** 10.1111/jop.13636

**Published:** 2025-04-11

**Authors:** Giuseppe Colella, Ciro Emiliano Boschetti, Francesca Farina, Maria Luisa Colella, Antonio Vicidomini, Nicola Cirillo

**Affiliations:** ^1^ Dipartimento Multidisciplinare di Specialità Medico‐Chirurgiche Ed Odontoiatriche University of Campania “Luigi Vanvitelli” Naples Italy; ^2^ Melbourne Dental School The University of Melbourne Melbourne Victoria Australia

**Keywords:** cancer treatment, hyaluronic acid, oral mucositis

## Abstract

**Introduction:**

It has been suggested that hyaluronic acid can prevent oral mucositis in patients receiving cancer treatment. We aimed to compare the efficacy of a commercial preparation of sodium hyaluronate spray supplemented with a pool of amino acids (Mucosamin) to standard oral care (SOC) in the prevention of chemotherapy‐induced oral mucositis.

**Methods:**

In this retrospective cohort study, consecutive cancer patients who had received chemotherapy without prior or concomitant radiotherapy at a tertiary care University Hospital in Naples, Italy, were eligible for inclusion. The exposure of interest was the use of Mucosamin spray prior to commencing the prescribed cycle of chemotherapy. The primary endpoint of the study was the onset of oral mucositis in both groups during and after their chemotherapy regimens. The secondary endpoint was to determine the severity of pain for patients who developed oral mucositis in either group.

**Results:**

A reduction was observed in the incidence of mucositis in patients who had used Mucosamin prophylactically (4%, 2/57) compared to SOC (33%, 16/49), with an effect size *ϕ* of 0.39. Patients in the Mucosamin group experienced a significant risk reduction (RR = 0.11; 95% CI 0.03–0.44). The use of Mucosamin resulted in an absolute risk reduction of 29.14%, and the number needed to treat to prevent one additional case of mucositis was 3.43.

**Discussion:**

Our results show that cancer patients receiving Mucosamin spray prior to the commencement of chemotherapy are at a reduced risk of developing oral mucositis compared to those receiving care as usual.

## 1 Introduction

Mucositis is a common complication of systemic high‐dose chemotherapy and radiation‐based anticancer treatments. The condition is characterized by inflammation and ulceration of the mucous membranes lining the alimentary canal, particularly in the mouth (oral mucositis, OM) and the gastrointestinal tract. Patients affected by OM may experience severe pain, difficulty swallowing, taste changes, decreases in weight, and secondary infections. When severe, OM can result in therapeutic noncompliance or can become a dose‐limiting toxicity that requires treatment modifications or interruption [1].

Despite being a predictable and hence potentially preventable condition, there is little to offer to patients who undergo cancer treatment to avoid this side effect [1, 2]. Hence, the development of prophylactic tools for mucositis is an unmet clinical need.

We have shown previously that hyaluronic acid‐based compounds can prevent or mitigate mucositis induced by cancer treatment in the laboratory [3] and in a small case series [4]. As Mucosamin has been used prophylactically in our unit for over 10 years in patients undergoing cancer treatment, we retrospectively assessed the risk of developing OM in patients receiving chemotherapy who were given this compound compared to care as usual.

## 2 Methods

Data of interest for this nested retrospective cohort study were identified from a database and retrieved retrospectively from outpatient hospital notes from the Department of Precision Medicine and the Oral and Maxillofacial Surgery Unit, University of Campania “Luigi Vanvitelli”, relevant to the period between January 2013 and December 2022.

Individuals aged 18 years and above with a histopathologically confirmed diagnosis of cancer and who were scheduled to receive chemotherapy, without concomitant radiotherapy, were eligible. Individuals with a previous history of cancer, who had undergone radiotherapy or had previously developed signs or symptoms of oral mucositis were excluded.

Exposures were standard recommendations, including basic oral care advice according to standard practice at the time [5] (care as usual/SOC) with or without Mucosamin spray. Follow ups were assessed for up to 6 months after the end of treatment.

The primary outcome was the onset of OM (WHO Oral Mucositis Grading Scale) [6] throughout the observation period. All patients developing mucositis were placed on routine treatment; therefore, only the first episode of OM was computed and analyzed for this endpoint. The secondary outcome was the subjective pain score (0–100) in patients developing OM. Statistical analysis was performed using SPSS and GraphPad Prism version 8.0.1 for Macbook (GraphPad Software Inc., San Diego, CA). Descriptive statistics are presented as means ± SD for continuous variables or frequencies and percentages for categorical variables.

Differences in the incidence of OM were analyzed using the Fisher's exact test (due to contingency tables with cell counts below five). Effect size was calculated using the Phi coefficient based on OM onset as a dichotomous variable (classified as small, medium, or large according to thresholds of 0.1, 0.3, and 0.5, respectively).

The Shapiro–Wilk test was used to assess the normality of continuous variables, such as chemotherapy‐induced oral mucositis (OM) pain scores. For normally distributed data, comparisons between groups were made using two‐tailed Student's unpaired *t*‐test.

Number needed to treat (NNT) was calculated based on Absolute risk reduction (ARR) as NNT = 1/ARR.

## 3 Results

Out of the 203 patients included in the database, 106 were eligible for the study; of these, 49 patients received standard care recommendations whereas 57 received Mucosamin before the first cycle of chemotherapy. Baseline characteristics of the patients, cancer diagnoses, and treatment received are summarized in Table 1.

**TABLE 1:** Patient demographics, cancer and treatment types, and cases of mucositis in the study cohort.

		Care as usual		Mucosamin	
		No.	%	No.	%
No. of patients		49	100	57	100
Sex	Female	38	78	37	65
	Male	11	22	20	35
Age (mean ± SD)		60 ± 12		59 ± 13	
Type of cancer	Breast	33	67	29	51
	Head & neck	2	4	7	12
	GIT	9	18	11	19
	Other	5	10	10	18
Therapy type	1st line	17	35	15	26
	2nd line	3	6	5	9
	3rd line	1	2	1	2
	Adjuvant	27	55	24	42
	Neoadjuvant	0	0	7	12
	Unlisted	1	2	5	9
Incidence of OM		16	33	2	4
RR (95% CI)	0.11 (0.03–0.44)				
Fisher exact test	*p* = 0.0001				

Abbreviations: CI, confidence interval; OM, oral mucositis; RR, risk ratio.

A significant reduction (*p* = 0.0001) was observed in the incidence of OM in patients who had used Mucosamin prophylactically (4%, *n* = 2) compared to the SOC group (33%, *n* = 16), with a medium effect size (*ϕ* = 0.39). Patients in the Mucosamin group experienced a significant risk reduction (RR = 0.11; 95% CI 0.03–0.44). The use of Mucosamin resulted in an ARR of 29.14%, and the NNT to prevent one additional case of mucositis was 3.43. After controlling for age, sex, and type of chemotherapy, only Mucosamin use was found to significantly (*p* < 0.005) affect the outcome.

**FIGURE 1:**
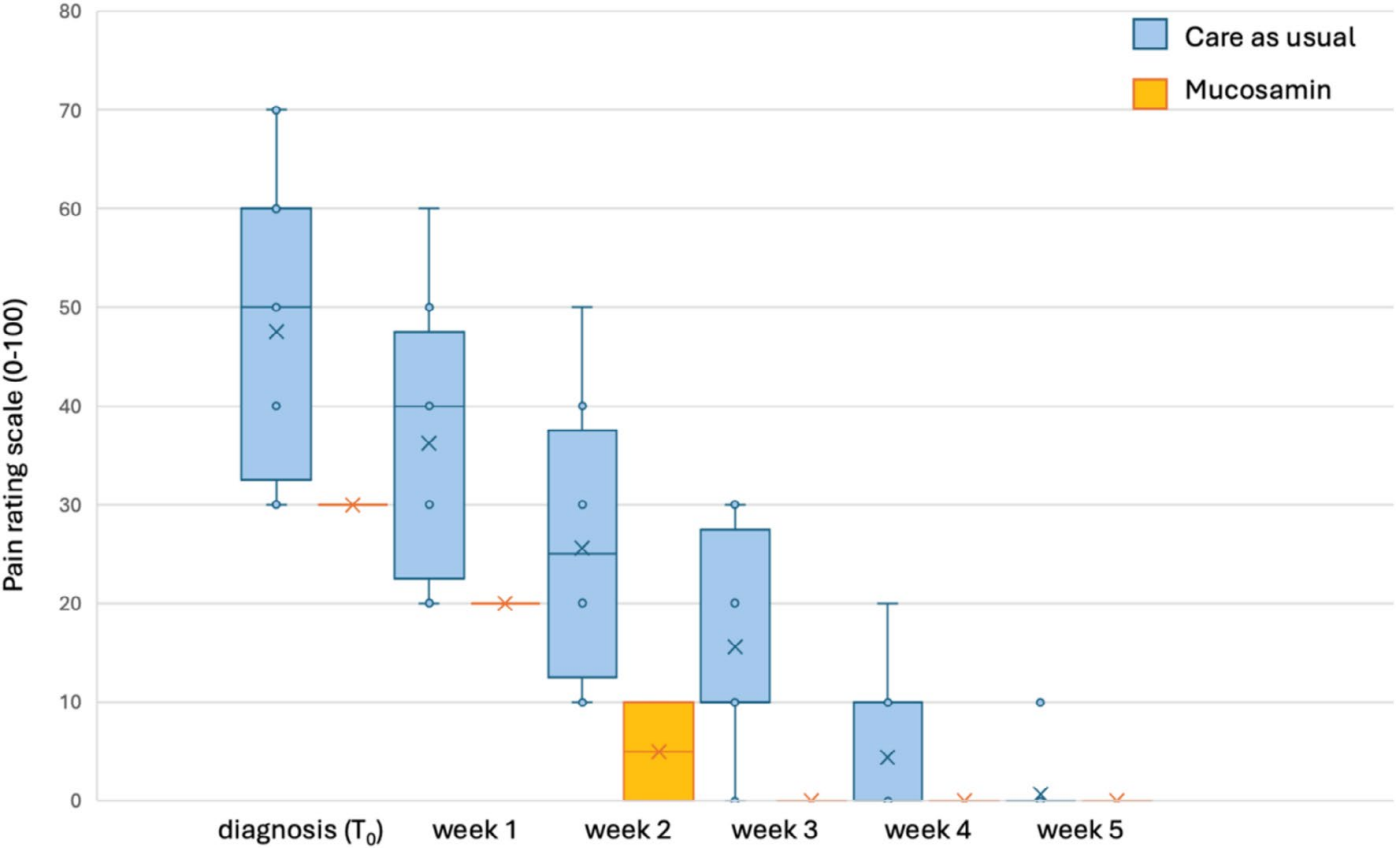
Average pain score for chemotherapy‐induced OM patients in Mucosamin spray group and control group from initial onset to 4 weeks after onset. Box plots depict interquartile range, mean, and data points. Note that patients developing OM were given Mucosamin spray three times daily until resolution of symptoms regardless of the preventive recommendations they received.

The chemotherapy‐induced OM pain scores rated by patients who received Mucosamin spray were generally lower than that of the patients who did not receive treatment (Table S1). No significant difference was noted between average pain scores rated by the two groups of patients at baseline (*p* = 0.1023; 95% CI −3.90 to 38.90) and on week 1 (*p* = 0.09; 95% CI −3.12 to 35.62), whereas pain was significantly lower for the Mucosamin group on week 2 (*p* = 0.04; 95% CI 0.98–40.27) There was no pain reported in the Mucosamin group from week 3 onwards (Figure 1).

## 4 Discussion

This retrospective cohort study shows that patients who used Mucosamin prophylactically as part of their oral care had a significantly lower risk of developing OM compared to those receiving standard care.

Given that mucositis is induced by cancer treatment and therefore is ultimately a predictable and potentially preventable condition, it is surprising that most research has been focused on treatment rather than prevention. The use of hyaluronic acid in the management of OM was first published over two decades ago [7], and later proposed for prevention [4].

Recent reports indicate that Mucosamin spray significantly reduces the severity of radiation‐induced OM in patients with oral squamous cell carcinoma [8]. A randomized controlled trial confirmed the effectiveness of a hyaluronic acid‐based mouthwash in preventing OM in pediatric patients undergoing hematopoietic stem cell transplantation [9]. This current study is the first to demonstrate the efficacy of Mucosamin in a hospital‐based adult population receiving chemotherapy for cancer.

It must be noted that this was an observational study derived from a secondary analysis of a prospectively collected database, and therefore prone to biases, particularly selection, observer, and recording bias. Additionally, we may not have fully accounted for confounding effects related to patients' factors. It is also important to underline that Mucosamin is a combination of sodium hyaluronate and essential amino acids, so we cannot definitively determine whether the observed effects were due to the former, the latter, or a combination of both. Nevertheless, the protective effects of the compound were so dramatic that the device warrants consideration for the prevention of mucositis.

In summary, as hyaluronic acid is a biocompatible natural constituent of connective tissues, the findings of this study could contribute to expanding the range of safe preventive measures available for OM in cancer patients.

## Author Contributions

Conceptualization, investigation, writing – review and editing, supervision, resources: G.C. Visualization, writing – review and editing: C.E.B., F.F., and M.L.C. Investigation, formal analysis, data curation: A.V. Conceptualization, methodology, formal analysis, writing – original draft, supervision, funding acquisition: N.C.

## Ethics Statement

The study protocol was approved by the Ethics Review Board of Azienda Universitaria Policlinico, Naples (no. 00165/2011). This study was conducted in full accordance with the ethical principles outlined in the Declaration of Helsinki.

## Conflicts of Interest

A.V. was in receipt of a bursary from Errekappa Euroterapici S.p.a. G.C. and N.C. served as consultants for Errekappa Euroterapici S.p.a.

## Supporting information


**Table S1.** Pain score for chemotherapy‐induced oral mucositis patients in Mucosamin spray group and control group from initial onset to 4 weeks after onset.

## Data Availability

The data that support the findings of this study are available in the Supporting Information of this article.
